# A bidirectional relationship between cognitive reserve and cognition among older adults in a rural Chinese community: a cross-lagged design

**DOI:** 10.3389/fpsyg.2023.1297699

**Published:** 2023-12-20

**Authors:** Hao Chen, Zhiyue Jiang, Jin Hu, Xing Yang, Shiqi Gui, Qiushuo Li, Jing Wang, Jingyuan Yang

**Affiliations:** ^1^Department of Epidemiology and Health Statistics, School of Public Health, The Key Laboratory of Environmental Pollution Monitoring and Disease Control, Guizhou Medical University, Guiyang, China; ^2^The Third People's Hospital of Guizhou Province, Guiyang, China; ^3^School of Medicine and Health Management, Guizhou Medical University, Guiyang, China

**Keywords:** cognitive reserve, older adults, latent profile analysis, confirmatory factor analyses, cross-lagged panel model

## Abstract

**Background:**

The concept of cognitive reserve (CR) plays a crucial role in understanding cognitive aging and resilience. Accumulating evidence revealed the influence of CR proxy on cognitive function, but it remains unknown whether a reverse association or reciprocal effect exists.

**Objective:**

The aim of this study is to observe the bidirectional relationship between cognitive reserve proxies and cognitive function among older adults in rural Chinese communities, providing a better understanding of the underlying mechanisms and potential moderating factors involved.

**Method:**

This longitudinal study analyzed 792 older adults (70.23 ± 5.87 years; 59.8%female) aged 60 years and older from the health status of rural older adults (HSRO) study over a 3-year period. Cognition was assessed by the Mini-Mental State Examination (MMSE). Cross-lagged panel modeling was utilized to analyze the interrelationship between cognitive reserve proxies and cognitive performance. Additionally, latent profile analysis was employed to identify different subtypes of neuropathic load within the study population.

**Results:**

Our cross-lagged analyses revealed significant associations between CR at T0 and MMSE scores at T1 (*β* = 0.81), as well as between MMSE scores at T0 and CR at T1 (*β* = 0.04). However, when conducting stratified analyses, we found no significant lagged relationships among individuals with high neuropathic load or those at an advanced age (*p* > 0.05). Furthermore, our longitudinal comparisons indicated changes in the contribution of CR proxy factors over time.

**Conclusion:**

The findings suggested a bidirectional relationship between cognitive reserve and cognitive performance in older adults. These results emphasized the importance of implementing timely public health measures to enhance cognitive reserve and cognitive performance ultimately promoting healthier aging among older adults.

## Introduction

With the aging of the population, cognitive impairment and dementia are prevalent among the elderly (Nichols et al., 2019). While there is currently no cure for dementia, the importance of prevention strategies is gaining recognition, with mounting evidence suggesting their potential to mitigate risk (Livingston et al., 2020). Cognitive reserve, an individual’s ability to withstand age-related changes and preserve cognitive function, is a key role that holds promise in this pursuit (Stern, 2012). While it is true that cognitive reserve is not directly observable or measurable, researchers have employed various proxy measures to capture its underlying mechanisms and effects (Jones et al., 2011; Xu et al., 2019).

Stern and colleagues (Stern et al., 2020) have argued that CR is a model of positive reserve that can change with lifestyle choices and events across the lifespan, improving the efficiency or flexibility of cognitive processes to cope with cognitive decline. Yet, it is not clear whether CR can accumulate across the lifespan, and future research should consider not only healthy lifestyle factors, but also the negative effects of cognitive aging (Oosterhuis et al., 2023). Previous research has focused on the unidirectional relationship, where CR proxies are considered to enhance cognitive reserve, which in turn supports better cognitive function (Stern, 2012; Mondini et al., 2016). Specifically, engaging in mentally stimulating activities or maintaining strong social connections may contribute to the enhancement of cognitive reserve, fostering resilience against cognitive decline. However, it remains unclear whether a reverse association is established. A perspective acknowledges that individuals with higher cognitive reserves may be more likely to actively seek out and participate in activities that stimulate their cognitive abilities (Kremen et al., 2022). Moreover, some studies have indirectly suggested that cognitive functioning might impact on cognitive reserve (Aartsen et al., 2002; Jones et al., 2011). In a study by Small et al. (2012), those who have poor cognitive function may also abandon several lifestyle activities and resulted for cognitive function decline. Within this context, it is important to contemplate an alternative perspective: individuals with higher levels of cognition proactively participate in activities that stimulate their cognitive abilities, potentially augmenting their cognitive reserve. This perspective prompts speculation about a potential bidirectional influence between cognitive reserve and cognitive function. However, a comprehensive investigation of this aspect is limited. Understanding the reciprocal influences between cognitive reserve and cognitive functioning is essential for gaining a comprehensive understanding of the dynamic interplay between these constructs.

In recent years, researchers have used two-way longitudinal studies to explore the causal priority relationships between various variables (Wang et al., 2017). The cross-lagged design, a form of structural equation modeling, estimates correlations between variables by analyzing data collected at two or more time points (Selig and Little, 2012; Kearney, 2017). This research approach allows us to examine the temporal order of the variables of interest, providing insight into how changes in one variable relate to subsequent changes in another variable over time. This temporal perspective is key for understanding the bidirectional relationship between cognitive reserve and cognitive functioning and determining the direction of influence.

In this study, we aimed to further explore the relationship between cognitive reserve and cognitive functioning in older adults through cross-lagged analyses and to elucidate the direction of influence. Thus, this is the first prospective study to explore the bidirectional relationship between cognitive reserve and cognition. We hypothesized that the relationship between CR and cognition may be bidirectional. Estimating the relationship between cognitive reserve and cognitive function is important for promoting healthy aging and developing interventions to optimize cognitive well-being.

## Methods

### Study design and participants

The cohort for this study was drawn from the Guizhou Rural Older Adults’ Health Study (HSRO) in China. The HSRO is a population-based prospective study conducted in Guizhou, China, utilizing a multistage cluster sampling approach. A total of 12 villages were selected, and the baseline survey took place from July to August 2019. Eligible participants were community volunteers aged 60 years or older, residing in the area for a minimum of 6 months. The study employed a two-wave (T0-T1) longitudinal survey design, with 1,654 older adults included in the assessment of cognitive reserve-related proxy measures at baseline. In 2021 (T1), a total of 792 individuals participated in the follow-up surveys. Ethical approval for the study was obtained from the Ethics Committee of Guizhou Medical University, and all participants provided informed consent.

### Measurement

#### Cognitive reserve

Cognitive reserve is a theoretical framework aimed at understanding the protective factors contributing to cognitive abilities. In our study, we collected data on four proxies of cognitive reserve: years of education, social support, hobbies, and exercise. Education was measured by a single item that asked participants to report the total number of years of schooling. Social support was assessed using the Social Support Rating Scale (SSRS) developed by Xiao (1994), which consisted of 10 items capturing subjective support, objective support, and support utilization. Responses to seven questions were measured on a four-point Likert scale, while the remaining questions involved calculating the number of support sources. Hobbies were assessed using a questionnaire comprising 10 items, including an item for indicating the absence of hobbies (Zheng, 2020). The number of hobbies was determined by assigning scores to the remaining items. We employed a single-item measurement method to assess exercise. The questionnaire’s exercise component was designed based on common durations of 30 and 60 min for the elderly population (Wang et al., 2022). Participants were asked to indicate their daily exercise time using response options ranging from “never” to “more than 60 min.” To analyze these variables, we employed confirmatory factor analysis, which enabled the construction of a latent variable model representing cognitive reserve.

#### Cognition

The Chinese version of the Mini-Mental State Examination (MMSE) scale was used to evaluate individuals’ cognition (Folstein et al., 1975; Katzman et al., 1988). The test includes 11 items, and the scores can immediately reflect global cognition in clinical, research, and community settings. The scores range from 0 to 30.

#### Covariates

Socio-demographic data were obtained for all populations. We used an enzyme-linked immunosorbent assay kit to quantify the levels of amyloid β-42 (E-El-H0543c, Elabscience, Wuhan, China) and phosphorylated tau protein (E-El-H5314c, Elabscience, Wuhan, China) in serum at baseline in the key laboratory of environmental pollution monitoring and disease control, Guizhou Medical University, and followed the manufacturer’s instructions. The inclusion of these proteins as covariates in the study aimed to control their potential impact on association between cognitive reserve and cognitive function by stratified analysis.

### Statistical analysis

All analyses were conducted with the R software (R project version 4.3.0; package: *tidyLPA, Lavaan, semTools*) and significance level was set at *α* = 0.05. Frequency and median (Interquartile Range (IQR); or p25, p75) were used to describe demographic characteristics. Non-parametric tests were employed to analyze the data. The Wilcoxon test was utilized for within-group comparisons of continuous variables with repeated measures, such as comparing baseline and follow-up data within the same group.

Latent profile analysis (LPA) was performed with neuropathologic biomarkers. The following fitting metrics were used in this study to select the optimal number of profiles: the Bayesian Information Criterion (BIC), the Akaike Information Criterion (AIC), and the Bootstrap Likelihood Ratio Test (BLRT) for model comparisons. The LPA was performed using a BIC, an AIC, BIC, and an entropy. Lower BIC, AIC, and higher entropy indicate a better fit. The BLRT primarily compares models in the fit difference class between k − 1 and k. To assess classification accuracy, entropy is reported with values ranging from 0.0 to 1.0, with higher values indicating greater accuracy (Eppig et al., 2017).

Confirmatory factor analysis (CFA) was separately conducted for the baseline and follow-up assessments to evaluate the goodness of fit of the cognitive reserve proxy factor structure. Eight indicators of goodness of fit were examined, including Chi square/df, Root Mean Square Error of Approximation (RMSEA), Comparative Fit Index (CFI), Tucker-Lewis Index (TLI), Normed Fit Index (NFI), Incremental Fit Index (IFI), Akaike information criterion (AIC), and Bayes information criterion (BIC). The following cut-off criteria were used to assess the fit index: NFI > 0.90, IFI > 0.90, TLI > 0.90, CFI > 0.90, RMSEA<0.05, and Chi square/df < 5 (West et al., 2012). The factor scores for CR were derived using the maximum likelihood method. A cross-lagged panel model was designed to examine the relationship between CR factor scores and cognitive scores at baseline and 3-year follow-up. This distinctive method allows the simultaneous analysis of two causal outcomes, identifying possible bidirectional associations over time. Moreover, it enriched our understanding of the intricate dynamics between cognitive reserve and cognitive function (Selig and Little, 2012). Model fit was considered using a selection of fit indices and criteria as above CFA.

## Results

The present study included a sample of 792 participants who completed the follow-up survey. The average age of the participants was 70.23 years (SD = 5.87). The sample consisted of 318 males and 474 females, providing a balanced representation of both genders. Among the participants, 96.7% had a sole occupation as farmers, indicating the predominance of this occupation in the rural Chinese community under investigation. Furthermore, 92.6% of the participants had received less than 6 years of education, highlighting the low educational attainment in this population (Supplementary Table S1). Latent profile analysis tested models for one to three profiles using Tau protein and beta amyloid as input variables. Model selection was based on the currently most recommended statistical model fitting criteria. The best models selected included solutions with two classes (Supplementary Table S2; Supplementary Figure S1). The participants in our study were categorized into two distinct profiles based on their levels of neuropathic load: high levels of neuropathic load and low levels of neuropathic loads. This classification allowed us to differentiate between individuals with significant neuropathic burden and those with comparatively lower levels of neuropathic indicators (Supplementary Table S3). We conducted a confirmatory factor analysis of the latent variable model for CR, demonstrating a good fit index at both baseline (T0) and follow-up (T1). Model fit indices are detailed in Supplementary Table S4 and Supplementary Figure S2. The measurement invariance of the CR model across time points was assessed using constraints. The results indicated that the model achieved configuration invariance and metric invariance (Δ(Metric – Configural model): Δχ^2^ = 2.28; Δ*df* = 3; ΔRMSEA = -0.012; ΔCFI = 0.003; ΔTLI = 0.028), suggesting that the measurement properties of the CR model were consistent over time (Supplementary Table S5).

**Figure 1:**
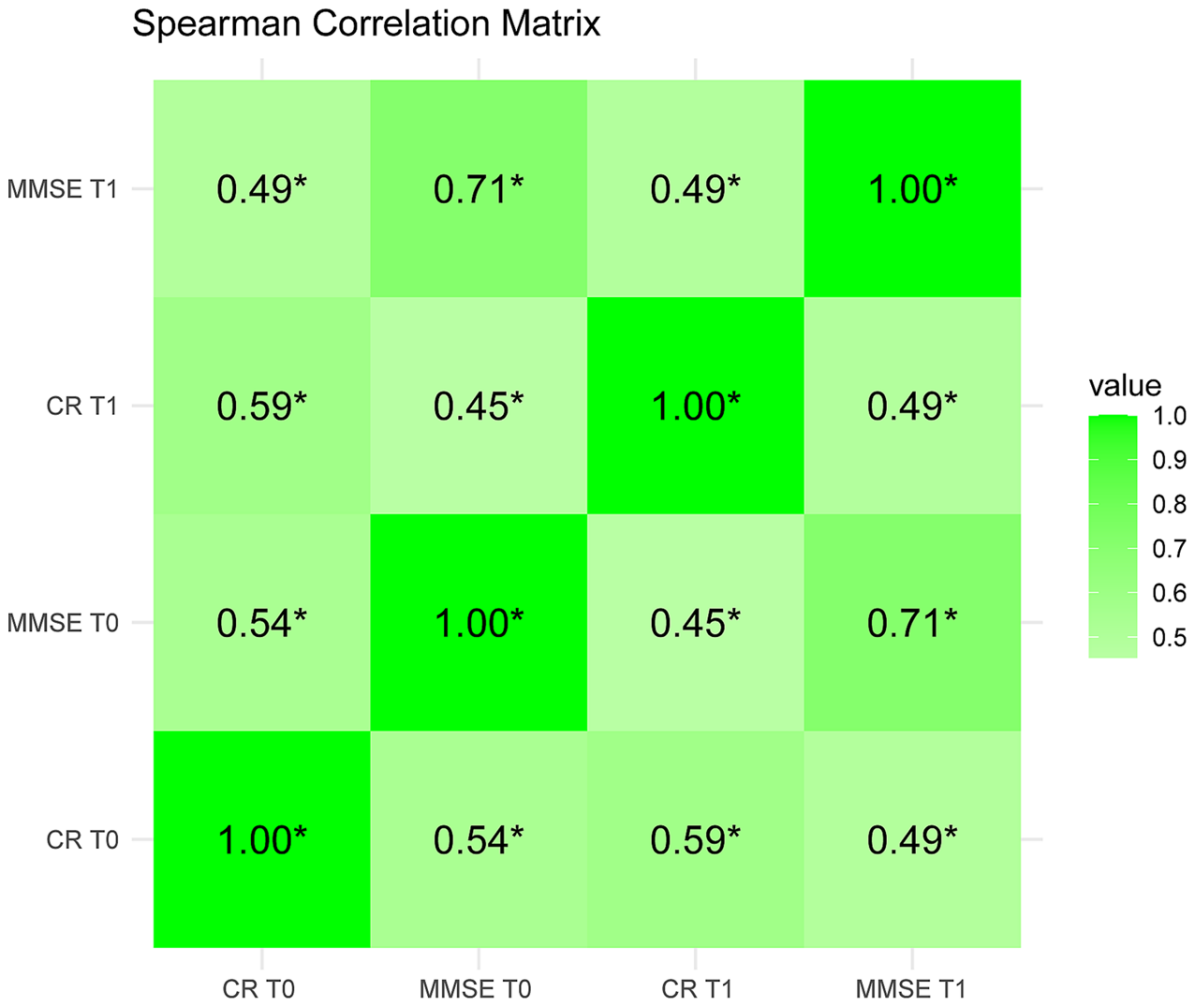
Correlation coefficients of two-wave CR and MMSE. T0 for bassline, T1 for follow-up. *denotes statistical significance, with *p<0.05 (two tailed).

In the longitudinal analysis of the demographic distribution (Table 1), a significant difference was observed in the distribution of MMSE scores between the baseline and follow-up assessments (*p* < 0.05). However, when examining the CR factor scores, there were no statistically significant differences in the longitudinal distribution of the other characters variables (*p* > 0.05), except for a significant difference in CR factor scores between the initial and follow-up assessments for females (*p* < 0.05). These findings indicate that while changes were observed in MMSE scores over time, the CR factor scores at T0 and T1 remained relatively stable (*p* > 0.05). As shown in Figure 1, there was a significant correlation between the baseline (CR0, MMSE0) and the follow-up measurements (CR1, MMSE1). All the correlations between variables were also significant.

**Table 1:** Longitudinal distribution of CR score, MMSE score.

Characteristic	Baseline (T0)	Follow-up (T1)	*Z*	*p* value
Total (*n* = 792)				
CR score, median (P_25_, P_75_)	−0.14(−0.68,0.59)	−0.06(−0.67,0.65)	−0.51	0.61
MMSE score, median (P_25_, P_75_)	22(18,26)	21(16,25)	−10.35	< 0.001
Female (*n* = 474)				
CR score, median (P_25_, P_75_)	−0.44(−0.91,0.18)	−0.28(0.91,0.33)	−2.14	0.03
MMSE score, median (P_25_, P_75_)	20(17,24)	19(15,22)	−8.48	< 0.001
Male (*n* = 318)				
CR score, median (P_25_, P_75_)	0.34(−0.24,0.99)	0.29(−0.32,1.02)	−1.71	0.08
MMSE score, median (P_25_, P_75_)	25(21,28)	24(19,27)	−5.97	< 0.001
Age group				
60–69(Years, *n* = 393)				
CR score, median (P_25_, P_75_)	−0.01(−0.59,0.65)	0.08(−0.58,0.83)	−1.02	0.31
MMSE score, median (P_25_, P_75_)	23(19,27)	22(17,26)	−7.18	< 0.001
70–79(Years, *n* = 344)				
CR score, median (P_25_, P_75_)	−0.18(−0.70,0.60)	−0.11(−0.75,0.58)	−0.15	0.88
MMSE score, median (P_25_, P_75_)	21(17,25)	20(16,24)	−6.28	< 0.001
≥80(Years, *n* = 55)				
CR score, median (P_25_, P_75_)	−0.36(−0.90,0.19)	−0.60(−1.12,0.21)	−1.00	0.32
MMSE score, median (P_25_, P_75_)	19(15,24)	15(10,21)	−4.27	< 0.001
High neuropathology load (*n* = 106)				
CR score, median (P_25_, P_75_)	−0.02(−0.66,0.64)	−0.14(−0.72,0.66)	−1.24	0.22
MMSE score, median (P_25_, P_75_)	22.50(18,26)	21(17,25)	−2.61	0.01
Low neuropathology load (*n* = 511)				
CR score, median (P_25_, P_75_)	−0.15(−0.70,0.64)	−0.04(−0.65,0.70)	−1.03	0.30
MMSE score, median (P_25_, P_75_)	22(18,26)	20(16,25)	−9.72	< 0.001

**Figure 2:**
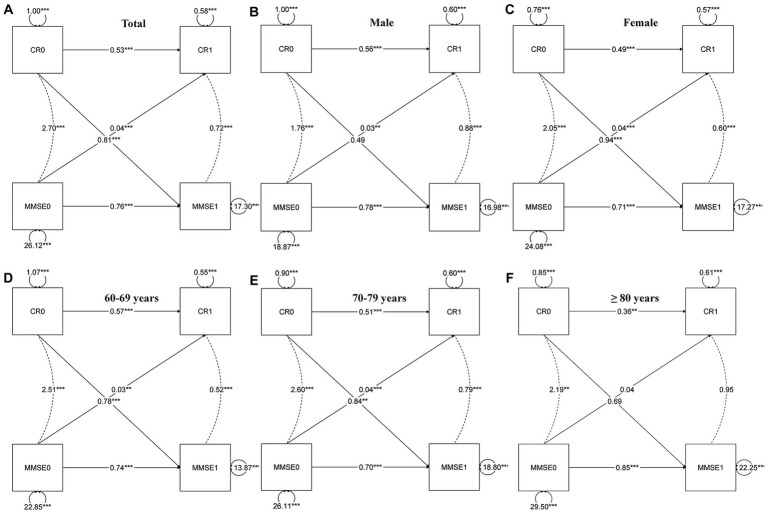
Cross-lagged model of CR and MMSE two-wave. Total **(A)**, gender **(B,C)**, and age group **(D–F)**; CR, cognitive reserve; MMSE, Mini-Mental State Examination. The notation of 0 and 1 to the right indicates T0 and T1, respectively, representing different time points in the study. All parameters presented are standardized path coefficients, *p<0.05 (two tailed); **p<0.01 5 (two tailed); ***p<0.001 (two tailed).

**Figure 3:**
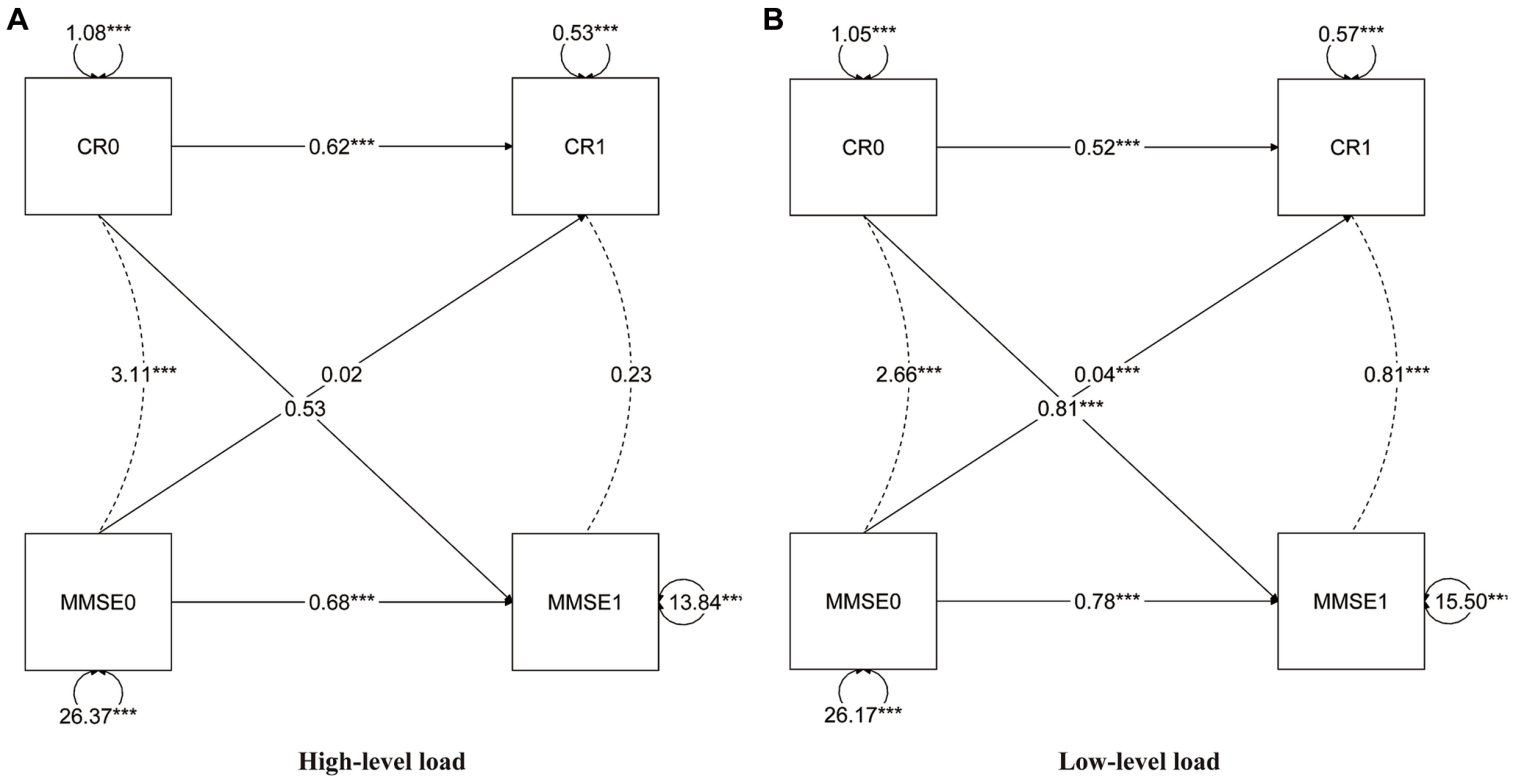
Cross-lagged model of CR and MMSE two-wave, neuropathology group. High neuropathic burden **(A)**, low neuropathic burden **(B)**. CR, cognitive reserve; MMSE, Mini-Mental State Examination. The notation of 0 and 1 to the right indicates T0 and T1, respectively, representing different time points in the study. All parameters presented are standardized path coefficients, ***p<0.001 (two tailed).

In the cross-lagged model (Figure 2), the autoregressive paths revealed carryover effects between CR scores and MMSE scores across different time points. The model fit the data well (RMSEA = 0.000; CFI = 1.000; TLI = 1.010). The parameter estimates provide evidence of bidirectional relationships within the model. Specifically, the current CR or MMSE (T1) received the autoregressive and lagged effects from the previous CR or MMSE (T0). For instance, the autoregressive path coefficient between CR1 and CR0 was estimated to be 0.53, indicating a significant influence of CR at the previous time point on CR at the current time point. Similarly, the cross-lagged path coefficient between MMSE1 and CR0 was estimated to be 0.81, demonstrating a significant influence of CR at the previous time point on MMSE at the current time point. Most of the coefficients of regression were statistically significant (*p* < 0.001); however, some of the lagged coefficients were not statistically significant for males, those over the age of 80, or individuals with high neuropathological burden (Figure 3).

**Figure 4:**
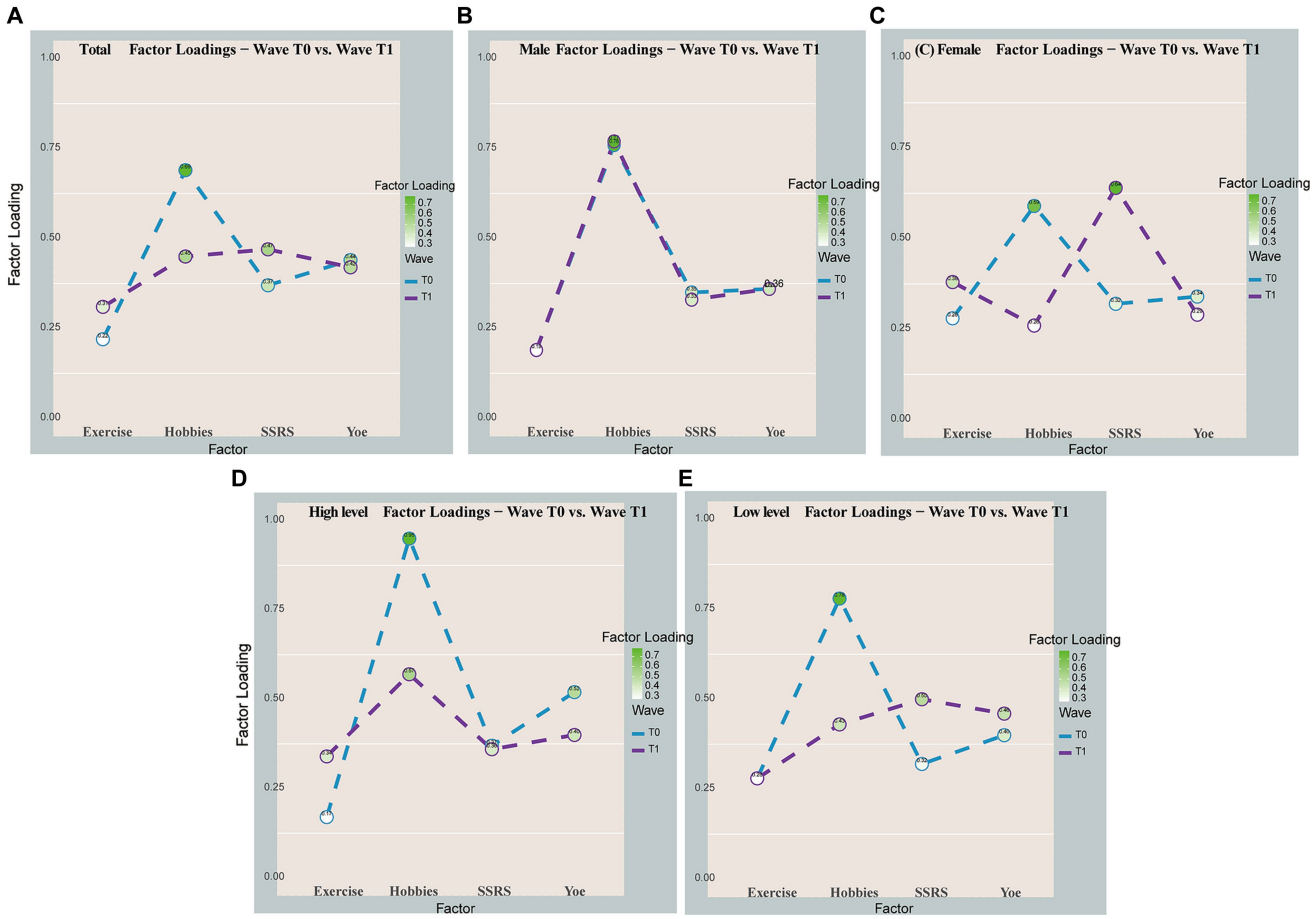
Visual longitudinal comparison of CR proxy factor loading changes. Total **(A)**, male **(B)**, female **(C)**, high-level neuropathology load **(D)**, and low-level neuropathology load **(E)**. The line graph was used to visualize the longitudinal variation of the factor loadings for only some of the subgroups.

In the total model with longitudinal comparisons of changes in CR proxy factor loadings (Table 2; Figure 4), the coefficient of loading on the contribution of CR proxy factors (social support) became larger longitudinally (0.37 to 0.47), while the coefficient of the hobbies became smaller (0.69–0.45). In the gender subgroup, male factor coefficients remained unchanged, while in the female subgroup, the factor coefficient of social support increased to its maximum level among the four factors, and the factor coefficient of hobbies decreased to its lowest level (Figure 4). At high levels of neuropathic burden, only the highest hobby loading coefficient (0.57) persisted as the most significant contributor, despite a longitudinal decrease, while the long-term loading coefficients for education (0.40), exercise (0.34), and social support (0.36) converged (Figure 4).

**Table 2:** Longitudinal comparison of changes in CR proxy factor loadings.

Wave	CR proxies			
	Yoe	SSRS	Hobbies	Exercise
	*β*-coefficient			
Total (*n* = 792)				
T0	0.44	0.37	0.69	0.22
T1	0.42	0.47	0.45	0.31
Female				
T0	0.34	0.32	0.59	0.28
T1	0.29	0.64	0.26	0.38
Male				
T0	0.36	0.35	0.76	0.19
T1	0.36	0.33	0.77	0.19
Age group				
60–69(Years)				
T0	0.42	0.31	0.75	0.24
T1	0.46	0.50	0.38	0.35
70–79(Years)				
T0	0.47	0.48	0.59	0.25
T1	0.30	0.52	0.41	0.32
≥80(Years)				
T0	—	—	—	—
T1	0.33	0.11	0.92	0.28
Neuropathology group				
High level load				
T0	0.52	0.37	0.95	0.17
T1	0.40	0.36	0.57	0.34
Low level load				
T0	0.40	0.32	0.78	0.28
T1	0.46	0.50	0.43	0.28

T0 = Time 0 (Baseline); T1 = Time 1 (Follow-up); All parameters were statistically significant (*p* < 0.05).

## Discussion

This study provided validation of the relationship between cognitive reserve and cognitive function among older adults in a rural Chinese community and was among the first to present local evidence on the bidirectional relationship between CR and cognition among older adults in this population with longitudinal data. Furthermore, our findings revealed a dynamic trend in the contribution of cognitive reserve proxies to the overall cognitive reserve, which enhances our comprehension of these intricate relationships over time.

As expected, our findings supported the notion that prior cognitive reserve proxies can indeed influence current cognitive performance. This aligns with a substantial body of previous research that has emphasized the critical role of cognitive reserve in maintaining cognitive function and resilience in the face of age-related decline (Stern, 2002, 2012; Pettigrew and Soldan, 2019). While the traditional perspective often emphasized the unidirectional influence of cognitive reserve proxies on cognition, it became increasingly evident that this relationship may be more nuanced. In our study, we observed that past cognitive abilities also exert an influence on current cognitive reserve proxies. This observation aligns with previous research indicated that cognitive abilities could shape social relationships (Wascher et al., 2018). While these researchers have contributed valuable insights, it’s important to note that their studies often offered a more isolated perspective, focusing on unidirectional relationships. In contrast, our study delves deeper into this complex interplay by exploring the bidirectional relationships between cognitive reserve and cognitive function. By directly examining the reciprocal influences, we not only corroborate prior evidence but also provide a more comprehensive understanding of the dynamics at play between these factors. This approach enriches the existing body of knowledge and highlights the multifaceted nature of cognitive reserve and its relation to cognitive function in older adults.

In addition, we also provided evidence that access to CR is beneficial for cognitive function in females, this has been consistent with the study by Subramaniapillai et al. (2021). Interestingly, our study revealed no bidirectional cross-lagged effect at high pathological conditions or high age (baseline≥80 years), perhaps because the threshold of cognitive resilience is exceeded due to being in the condition of two factors. Similarly, there is some evidence that CR’s protective effect may diminish with neuronal damage or cognitive disease processes (Mungas et al., 2018; Pettigrew and Soldan, 2019). By exploring this potential bidirectional relationship, our study aimed to provide valuable insights into the complex interplay between CR and cognition. A deeper understanding of these mechanisms is essential for fostering CR plasticity and developing effective interventions to enhance cognitive function.

As the cognitive reserve is plastic, it can be influenced by experiences at all stages of life. Focused research in this area can maximize the chances of successful interventions (Ward et al., 2015; Stern, 2021). Consequently, we conducted a further analysis to explore the longitudinal variation in the contribution of CR proxies. We found that a decreasing contribution of hobbies to cognitive reserve was observed over time, while the contribution of social support showed an increasing trend. Perhaps, the limited elderly care infrastructure in rural areas may have resulted in reduced impact from activity participation or hobbies among rural older adults (Liu et al., 2019). On the other hand, social support becomes increasingly important as older adults may rely more on social connections for cognitive stimulation, emotional well-being, and access to resources (Zahodne et al., 2019). Older adults may face increased challenges and transitions, such as retirement or loss of loved ones, making social support crucial for maintaining cognitive function. Also, the distinctive socio-cultural context of rural areas may introduce unique dynamics into the relationship between cognitive reserve and cognitive function within this population (Chen et al., 2022). These complex interactions among biological, lifestyle, and social factors likely contribute to the observed changes in the contribution of specific factors to cognitive reserve in older adults.

Furthermore, our study revealed interesting gender differences in the contribution of CR proxies. Among women, social support emerged as the most influential factor for cognitive reserve over time. In contrast, there was no clear change in the factor loadings for men. One explanation is that for women social engagement, more frequent contact, is stimulating for women, in contrast, among men this may be the role of being the head of the family in the home environment resulting in not much change in social relationship stimulation (Zunzunegui et al., 2003). In our study, we found that hobbies had the highest impact on cognitive reserve among individuals with high levels of neuropathological burden. However, we did not observe a lagged relationship between cognitive reserve and cognition, suggesting that the effects of hobbies on cognitive reserve may not have long-term implications for cognitive function, or that the presence of neuropathology may mediate this relationship.

This study has several limitations: First, it is important to note that the cognitive reserve proxies used in our study, while commonly employed, may not fully capture the cognitive reserve of the rural elderly population. This could limit the generalizability of our findings to other populations or settings. Second, the small sample size and limited number of follow-up visits among older adults may have impacted the statistical power of our analysis, potentially hindering our ability to capture dynamic changes in cognitive reserve over time. To obtain a more reliable assessment of the relationship between CR and cognitive outcomes, future studies should consider larger sample sizes and longer follow-up periods. Third, the cognitive-related biological indicators utilized in this study, although widely used, may not be comprehensive enough to fully represent the neuropathologic load. It is important to consider additional measures that provide a more accurate assessment of neuropathology. Forth, the measurement invariance did not pass the stricter invariance constraint. However, achieving full measurement invariance proved challenging, potentially influenced by sample changes or modifications in measures (Putnick and Bornstein, 2016). Studies in the cognitive domain have faced similar issues, with significant changes observed in intercepts and residuals over time, possibly due to sample-specific characteristics (Bertola et al., 2021). Lastly, the measurement of cognitive function using instruments such as the Mini-Mental State Examination is susceptible to measurement error and may have a ceiling effect, particularly in populations with high baseline cognitive performance. This limitation may restrict our ability to detect subtle changes in cognitive performance and impact the accuracy of observed correlations, potentially limiting our ability to fully assess cognitive reserve and its correlation with cognitive function. These considerations should be taken into account when interpreting our findings and highlight the need for further research with improved methodologies to enhance our understanding of cognitive reserve and its association with cognitive function.

In conclusion, by investigating the bidirectional relationship between cognitive reserve proxies and cognitive function in rural Chinese older adults, this study fills an important research gap and provides valuable insights into the factors influencing cognitive well-being in this specific population. The findings contribute to the existing literature by expanding our understanding of cognitive reserve in the context of rural Chinese communities and provide a foundation for developing targeted interventions that enhance cognitive reserve and promote healthy aging.

## Data availability statement

The raw data supporting the conclusions of this article will be made available by the authors, without undue reservation.

## Ethics statement

The studies involving humans were approved by the Ethics Committee of Guizhou Medical University. The studies were conducted in accordance with the local legislation and institutional requirements. The participants provided their written informed consent to participate in this study.

## Author contributions

HC: Investigation, Conceptualization, Methodology, Writing – original draft, Writing – review & editing. ZJ: Data curation, Writing – review & editing. JH: Investigation, Writing – review & editing. XY: Supervision, Writing – review & editing. SG: Investigation, Writing – review & editing. QL: Investigation, Writing – review & editing. JW: Investigation, Writing – review & editing. JY: Investigation, Methodology, Software, Writing – review & editing.
