# Supplementary material for: A bidirectional relationship between cognitive reserve and cognition among older adults in a rural Chinese community: a cross-lagged design
Source: Front Psychol. 2023 Dec 20;14:1297699. doi: 10.3389/fpsyg.2023.1297699 (PMC10773703; doi:10.3389/fpsyg.2023.1297699)
Supplement: Supplementary file 1 [file Table_1.DOCX]

Table S1 Distribution of socio-demographic information

| Characteristic | Baseline | Follow-up |
| --- | --- | --- |
|  | n=1654 | n=792 |
| Age, Y, mean (SD), | 71.36(6.57) | 73.62(5.84) |
| Male/Female, | 690/954 | 318/474 |
| Farmer, n (%) | 1510(91.3) | 766(96.7) |
| Education level, Y, n (%) |  |  |
| 0~ | 991(59.9) | 604(76.3) |
| 3~ | 303(18.3) | 129(16.3) |
| 6~ | 222(13.4) | 52(6.6) |
| 9~ | 138(8.3) | 7(0.9) |
| SSRS, median (IQR) | 38(9) | 37(10) |
| Exercise, hours, n (%) |  |  |
| Never | 928(56.1) | 289(36.0) |
| 0~ | 295(17.8) | 251(31.3) |
| 0.5h~ | 246(14.9) | 127(15.8) |
| 1h~ | 185(11.2) | 136(16.9) |
| Hobbies, n (%) |  |  |
| 0~ | 1496(90.4) | 230(29.0) |
| 3~ | 152(9.2) | 496(62.7) |
| 6~ | 6(0.4) | 66(8.4) |
| MMSE score, median (IQR) | 21(8) | 21(9) |

Note: SSRS, Social Support Rating Scale; MMSE, Mini-Mental State Examination.

Table S2 Fitting of latent profile analysis classes

| Classes | AIC | BIC | Entropy | BLRT_p |
| --- | --- | --- | --- | --- |
| 1 | 3507.94 | 3525.64 | 1.00 | - |
| 2 | 3176.47 | 3207.44 | 0.86 | 0.01 |
| 3 | 3182.59 | 3226.84 | 0.44 | 0.59 |

Note: AIC, Akaike’s Information Criterion; BIC, Bayesian Information Criteria

Table S3 Grouping of latent profile analysis of neuropathological indicators

| Neuropathology group | P-tau(pg/ml) | β_1-42_(pg/ml) |
| --- | --- | --- |
| High level load | 239.50±86.55 | 320.14±161.02 |
| Low level load | 71.01±47.32 | 105.50±75.689 |

Table S4 Goodness-of-Fit Statistics for T0 and T1 Confirmatory Factor Analysis Models

| Goodness-of-fit | T0(n=1654) | T1(n=792) |
| --- | --- | --- |
| *χ^2^/df* | 3.21/2 | 7.47/2 |
| RMSEA | 0.02 | 0.05 |
| Comparative Fit Index (CFI) | 0.99 | 0.96 |
| Tucker-Lewis Index (TLI) | 1.00 | 0.87 |
| Bentler-Bonett Normed Fit Index (NFI) | 0.99 | 0.94 |
| Bollen's Incremental Fit Index (IFI) | 1.00 | 0.96 |
| Akaike’s Information Criterion (AIC) | 24395 | 14662 |
| Bayesian Information Criteria (BIC) | 24460 | 14719 |

Table S5 Longitudinal measurement invariance of the CR model

| Model | χ^2^ | *df* | RMSEA | CFI | TLI | AIC | BIC | *P* |
| --- | --- | --- | --- | --- | --- | --- | --- | --- |
| Configural Invariance | 9.165 | 4 | 0.040 | 0.981 | 0.944 | 28388.806 | 28517.631 | _ |
| Metric Invariance | 11.451 | 7 | 0.028 | 0.984 | 0.973 | 28385.092 | 28497.814 | _ |
| Scalar Invariance | 97.637 | 9 | .112 | .681 | .575 | 28467.278 | 28569.264 | _ |
| Strict Invariance | 106.94 | 13 | .096 | .662 | .688 | 28468.581 | 28549.097 | _ |
| Δ(metric – config) | 2.2861 | 3 | -0.012 | 0.003 | 0.028 | -3.714 | -19.817 | 0.52* |
| Δ(scalar – metric) | 86.186 | 2 | 0.083 | -0.303 | -0.398 | 82.186 | 71.451 | <0.001 |
| Δ(strict – scalar) | 9.303 | 4 | -0.016 | -0.019 | 0.113 | 1.303 | -20.167 | 0.053 |

Notes: AIC,Akaike’s Information Criterion; BIC, Bayesian Information Criteria; CFI, comparative fit index; RMSEA, root mean square error of approximation; TLI, Tucker–Lewis index; * indicated an accepted model.


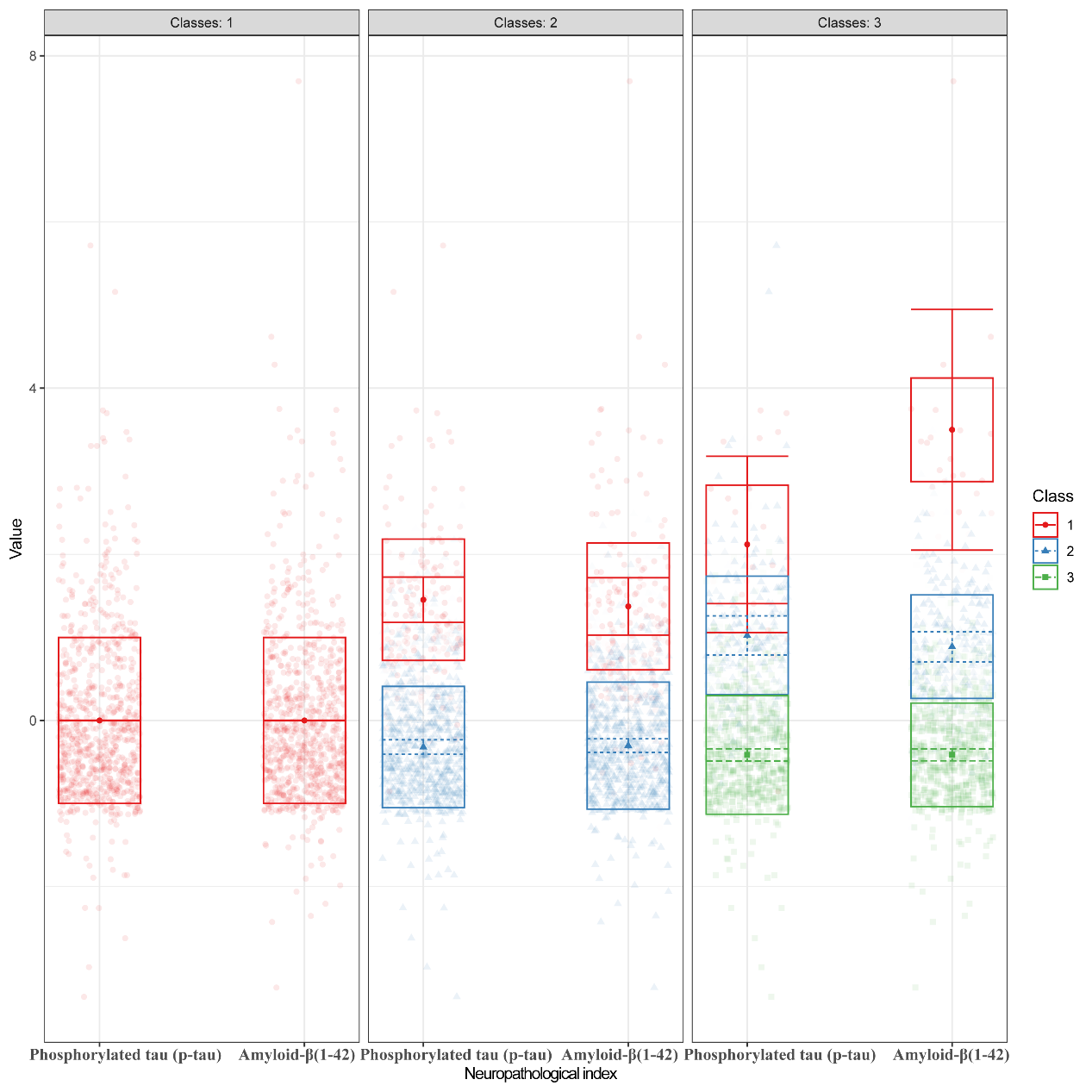


Figure S1 Visualization grouping for latent profile analysis in neuropathology


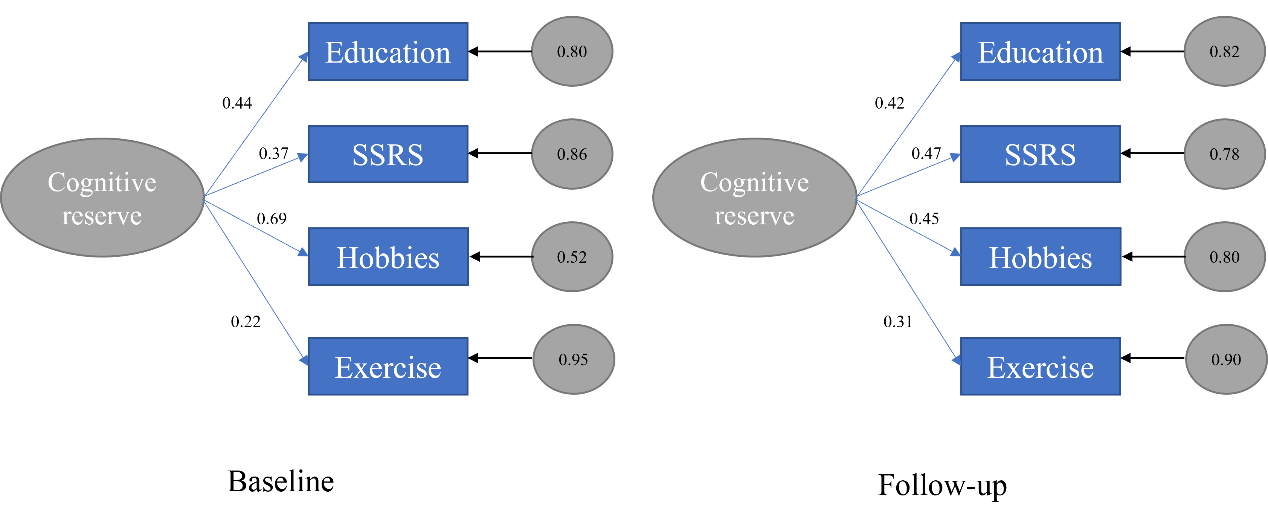


Figure S2 Confirmatory factor analysis of CR model
